# Periprosthetic Joint Infection in Unicompartmental vs. Total Knee Arthroplasty: Microbiological Spectrum and Management Outcomes

**DOI:** 10.3390/antibiotics14060585

**Published:** 2025-06-06

**Authors:** Ali Said Nazlıgül, Şahan Güven, Yasin Erdoğan, Ahmet Fırat, Metin Doğan, Mustafa Akkaya

**Affiliations:** 1 Department of Orthopaedics & Traumatology, Sincan Training and Research Hospital, 06949 Ankara, Türkiye; 2 Department of Orthopaedics & Traumatology, Ankara Bilkent City Hospital, 06800 Ankara, Türkiye; 3 Department of Orthopaedics & Traumatology, Etlik City Hospital, 06170 Ankara, Türkiye; 4 Department of Orthopaedics & Traumatology, Medical Park Ankara, 06680 Ankara, Türkiye; 5 Department of Orthopaedics & Traumatology, Yüksek İhtisas University, 06530 Ankara, Türkiye; 6 Department of Orthopaedics & Traumatology, Ankara Güven Hospital, 06100 Ankara, Türkiye

**Keywords:** periprosthetic joint infection, unicompartmental knee arthroplasty, total knee arthroplasty, microbiological spectrum

## Abstract

**Background/Objectives**: Periprosthetic joint infection (PJI) is a severe complication following both total knee arthroplasty (TKA) and unicompartmental knee arthroplasty (UKA). While the microbiological profile of TKA PJI has been well characterized, limited data exist regarding UKA PJIs. This study aimed to compare the causative microorganisms and surgical treatment outcomes in PJI cases following UKA and TKA. **Methods**: This retrospective cohort study included 82 patients (71 TKA and 11 UKA) who underwent surgical treatment for PJI between January 2017 and May 2024. PJI was diagnosed based on the Musculoskeletal Infection Society (MSIS) criteria. Treatment strategies included debridement, antibiotics, and implant retention (DAIR) or two-stage revision arthroplasty. Microbiological data were extracted from intraoperative cultures. Fisher’s exact test and the Mann–Whitney U test were used for statistical comparisons. **Results**: Gram-positive organisms, primarily *Staphylococcus aureus* and coagulase-negative staphylococci, were isolated in all UKA PJIs. In contrast, the TKA group demonstrated greater microbial diversity, including Gram-negative bacilli, polymicrobial infections, and a higher rate of culture-negative cases (33.8% vs. 18.2%). DAIR was performed more frequently in UKA cases (72.7% vs. 28.2%, *p* = 0.002). Recurrence rates following DAIR were similar in both groups (12.5% in UKA, 20.0% in TKA, *p* = 1.000). Two-stage revision resulted in no recurrence in UKA and a 9.8% recurrence rate in TKA patients. **Conclusions**: UKA PJIs appear to be microbiologically less complex than TKA PJI cases, with Gram-positive organisms predominating. Despite these differences, the outcomes of surgical treatment—both DAIR and two-stage revision—were comparable between groups. Standard PJI treatment principles may be applicable to both arthroplasty types; however, larger prospective studies are needed to confirm these findings.

## 1. Introduction

Unicompartmental knee arthroplasty (UKA) is an effective surgical procedure that improves quality of life in patients with isolated compartmental osteoarthritis [1,2]. In the past decade, national knee arthroplasty registries from the United Kingdom and the United States have reported a notable increase in the popularity of UKA, with UKA accounting for approximately 4% to 14% of all knee arthroplasty procedures [3,4].

UKA offers several important advantages over total knee arthroplasty (TKA). These include preservation of the cruciate ligaments, a movement pattern that more closely resembles normal knee kinematics, reduced blood loss, shorter hospital stay, and faster postoperative functional recovery [1,5]. In addition, smaller surgical incisions and shorter operative times may contribute to a reduced risk of infection. However, despite all these advantages, periprosthetic joint infection (PJI) remains a serious complication that can occur following UKA, just as it does after TKA. Such infections may lead to repeated surgical interventions and significant functional impairment [6].

The incidence of PJI following UKA has been reported to be significantly lower compared to TKA. In the literature, the incidence of PJI following UKA typically ranges from 0.3% to 1.3%, whereas this rate may reach up to 1.0–2.5% for TKA [7,8,9]. However, despite the difference in incidence, PJI following either procedure can have similarly devastating clinical consequences. Moreover, due to the relative rarity of UKA PJI, the clinical course, microbiological characteristics, and optimal treatment strategies remain limited and heterogeneous in the current literature. In contrast, TKA PJI has been better characterized, with numerous studies available on causative microorganisms, risk factors, and treatment outcomes [10,11].

From a microbiological perspective, the most common causative agents of TKA PJI are Gram-positive cocci [11]. However, Gram-negative bacilli and polymicrobial infections are also frequently encountered, which significantly complicates empirical antibiotic selection and treatment decision-making [11]. In contrast, the causative organisms of UKA PJI have not been well defined in the literature, and the limited number of available studies generally indicate a predominance of methicillin-sensitive staphylococcal species [6,12]. However, data on polymicrobial infections, Gram-negative bacilli, and resistant pathogens in UKA PJI cases are quite limited, and the available findings are mostly based on small case series [12,13]. Therefore, further studies are needed to elucidate the clinical and microbiological characteristics of UKA PJIs.

The surgical management of PJI mainly includes two approaches: debridement, antibiotics, and implant retention (DAIR) and two-stage revision arthroplasty [14]. DAIR, typically preferred in acute infections, involves debridement while retaining the implant. Two-stage revision, considered the gold standard for chronic infections, includes implant removal, insertion of an antibiotic-loaded spacer, and delayed reimplantation [15]. The choice between these strategies depends on infection chronicity, microbial characteristics, and patient-related factors [14,15].

The aim of this study is to compare the microbiological pathogens identified in PJIs following UKA and TKA, to evaluate potential differences in their distribution, and to assess the surgical treatment strategies used in these cases in light of current literature.

## 2. Results

The groups were compared in terms of demographic and clinical characteristics. A statistically significant difference was observed in mean age, with UKA patients being younger than TKA patients (*p* = 0.004). However, no significant differences were found between the groups in terms of sex distribution or operated side (*p* > 0.05 for both). Follow-up durations were also analyzed, with a median follow-up of 49 months in the UKA group and 45 months in the TKA group (Table 1).

In the TKA group, 20 patients underwent DAIR, while 51 patients were treated with two-stage revision surgery using an antibiotic-loaded spacer. Among those who underwent DAIR, 4 patients required subsequent two-stage revision due to failure of infection control, whereas the remaining 16 showed no recurrence during the follow-up period. In the UKA group, eight patients underwent DAIR, and three patients were treated with two-stage revision surgery using an antibiotic-loaded spacer. Of the eight DAIR-treated UKA patients, one experienced infection recurrence during follow-up and required two-stage revision surgery (Table 2).

The microbiological profiles differed between the two groups. In the UKA group, all infections were caused by Gram-positive organisms, predominantly *Staphylococcus aureus* and coagulase-negative staphylococci. No Gram-negative or polymicrobial infections were observed in this group. In contrast, the TKA group showed a broader spectrum of pathogens, including Gram-negative bacilli, Enterobacteriaceae species such as *Escherichia coli* and *Klebsiella* spp., as well as non-fermentative organisms like *Pseudomonas aeruginosa*. Polymicrobial infections were also present only in the TKA group. Culture-negative cases were more frequent in TKA than in UKA. There were 24 culture-negative cases (33.8%) in the TKA group and 2 cases (18.2%) in the UKA group (Table 3).

## 3. Discussion

PJI remains a devastating complication after both UKA and TKA. Although the microbiological characteristics of PJI in TKA are well described in the literature, data on the microbial etiology of PJI in UKA remain scarce. When PJI does occur, our comparative analysis demonstrated notable microbiological differences between UKA and TKA. All isolates in the UKA group were Gram-positive cocci, primarily methicillin-sensitive *Staphylococcus aureus* and coagulase-negative staphylococci, with no cases of Gram-negative or polymicrobial infections identified. In contrast, TKA PJIs exhibited a broader microbial spectrum, including Enterobacteriaceae (*Escherichia coli*, *Klebsiella* spp.), non-fermentative Gram-negative bacilli (*Pseudomonas aeruginosa*), and polymicrobial infections—features observed exclusively in the TKA cohort.

Staphylococci are the most frequently isolated pathogens in PJIs; in UKA PJI cases in particular, *Staphylococcus aureus* and other Gram-positive skin flora organisms are the predominant causative agents [16,17]. In contrast, although Gram-negative bacilli are less common than Gram-positive organisms, they account for approximately 10–15% of TKA PJI cases. Polymicrobial PJIs are typically observed in acute postoperative infections and constitute around 5–10% of TKA PJI cases [11,18,19]. In our study, Gram-negative organisms were identified in 11% of TKA PJI cases, and a small number of cases were polymicrobial; however, no such pathogens were detected in the UKA group. In contrast, Cavagnara et al., in their study of patients undergoing two-stage revision for UKA PJI, reported the presence of both polymicrobial and Gram-negative infections [20].

A difference in the frequency of culture-negative infections was also observed between the groups. In approximately one-third of TKA PJI cases, no microbial growth was detected despite a diagnosis based on MSIS criteria. In contrast, the culture-negative rate in the UKA group was found to be around 18%. According to the literature, the reported incidence of culture-negative PJI in TKA cases ranges between 10% and 40% [11,21,22]. However, in UKA PJI cases, this rate has been reported to range between 0% and 18% [12,13,23]. In our study, the higher rate of culture-negative results observed in the TKA group may be associated with the chronic nature of infections or prior antibiotic exposure, both of which can reduce the likelihood of isolating microorganisms in culture. In contrast, the lower culture-negativity rate in the UKA group may be related to the diagnosis of PJI being established in the acute phase.

In both groups, acute or acute hematogenous PJI cases were treated using the DAIR. In our study, among the eight patients in the UKA group who underwent DAIR, infection recurrence was observed in one patient (12.5%) during the follow-up period. McCormick et al. reported a 1-year infection control rate of 80.8% in acute UKA PJI cases treated with DAIR [12]. However, some studies have shown that long-term success rates following DAIR are lower. In a study by Asadollahi et al., only 57% of patients with acute infected UKA treated with DAIR remained infection-free at the five-year follow-up [13]. In the TKA group, DAIR was successful in 75% of the patients. Similarly, the literature reports that the success rate of DAIR in acute TKA PJI cases ranges between 60% and 80% [24,25,26]. In a systematic review performed by Longo et al. involving 970 patients, an average infection-free survival rate of 71% was reported following DAIR in acute hip and knee PJI cases [10]. Based on these findings and the results of our study, it can be concluded that UKA PJI cases demonstrate a similar response to DAIR treatment as TKA PJI cases. This indicates that, as with TKA, DAIR may serve as an effective and viable treatment option for acute UKA PJI when appropriate patient selection and surgical technique are applied. However, the limited number of UKA PJI cases may have restricted the statistical power for subgroup comparisons, particularly regarding treatment outcomes.

In the literature, the infection eradication rates following two-stage revision surgery for TKA PJI cases have been reported to range between 80% and 90% [27,28,29]. In our study, among the patients who underwent two-stage revision surgery, infection recurrence was observed in 5 out of 51 patients in the TKA group during the follow-up period. In contrast, no cases of infection recurrence were detected in the UKA group among those who underwent two-stage revision.

In our study, all UKA PJI cases were caused by Gram-positive organisms, with no Gram-negative or polymicrobial infections identified. While this finding suggests that initial empirical therapy may reasonably focus on Gram-positive coverage in UKA PJI, especially in resource-limited settings, clinicians should remain cautious. Gram-negative pathogens, although not observed in our cohort, have been reported in the literature [20,23]. Therefore, empirical regimens should be tailored to local epidemiology, patient risk factors, and institutional resistance profiles to avoid undertreatment.

In conclusion, Gram-positive microorganisms were found to be predominantly associated with UKA PJIs, whereas a broader microbiological diversity was observed in TKA PJI cases. This distinction may serve as a clinical guide, particularly in planning empirical antibiotic therapy. Although there are some microbiological differences between UKA and TKA PJIs, the treatment algorithms applied appear to yield similar clinical outcomes. Based on these findings, it can be suggested that the standard principles recommended for PJI management may largely be applied similarly to both UKA and TKA. However, the retrospective and single-center nature of this study, along with the limited UKA sample size, should be considered when interpreting these results. In addition, the absence of systematic data on patient comorbidities may also limit the interpretation of infection-related risk factors. To better define the distribution of isolated microorganisms in UKA PJIs and to further optimize treatment strategies, prospective studies with larger patient cohorts are needed.

## 4. Materials and Methods

Following approval from the institutional ethics committee (1-25-1252, 7 May 2025), we conducted a retrospective cohort study involving patients who underwent surgical treatment for periprosthetic joint infection after primary UKA or TKA at a tertiary care center between January 2017 and May 2024.

The institutional surgical archive was screened to identify patients with a diagnosis of PJI following UKA or TKA. The inclusion criteria were as follows: (1) diagnosis of PJI based on the Musculoskeletal Infection Society (MSIS) criteria, including clinical, laboratory, and intraoperative findings; (2) treatment with either DAIR or two-stage revision arthroplasty using an antibiotic-loaded cement spacer; and (3) available and complete microbiological records including intraoperative cultures. Exclusion criteria included revision arthroplasty prior to infection, culture results suggestive of contamination or insufficient data, and incomplete clinical follow-up documentation.

The study included 82 patients with PJI, comprising 11 UKA and 71 TKA cases. Patient demographics, including age, gender, and date of surgery, were obtained from institutional medical records. Surgical procedures were categorized as DAIR or two-stage revision with spacer placement. The distribution of surgical treatments was recorded separately for the UKA and TKA groups. PJI cases were classified as acute (including early postoperative and hematogenous infections) or chronic according to the Tsukayama criteria [30]. Acute and acute hematogenous infections were treated with DAIR, whereas chronic infections were managed with two-stage revision arthroplasty. Intraoperative culture results, including tissue and synovial fluid samples, were retrospectively reviewed based on microbiology reports. Culture-positive PJI was defined as the isolation of one or more organisms consistent with infection. If multiple distinct pathogens were identified, the case was classified as polymicrobial. Culture-negative PJI was defined as the absence of any pathogen despite strong clinical suspicion and intraoperative evidence of infection. Data on comorbidities were not systematically available across all patients and were therefore not included in the analysis.

### Statistical Analysis

Statistical analyses were performed using IBM SPSS Statistics version 25 (IBM Corporation, Armonk, NY, USA). Descriptive statistics for categorical variables were expressed as frequencies (*n*) and percentages (%), while continuous variables were presented as mean ± standard deviation (SD) or median (25th–75th percentiles), as appropriate. The normality of the distribution of continuous variables was assessed using the Kolmogorov–Smirnov test, and the assumption of homogeneity of variances was evaluated using Levene’s test. Group comparisons for continuous variables were conducted using the independent samples *t*-test when normality assumptions were met; otherwise, the Mann–Whitney U test was applied. For categorical variables, the chi-square test or Fisher’s exact test was used as appropriate. Unless otherwise stated, a *p*-value of <0.05 was considered statistically significant.

## Figures and Tables

**Table 1:** Demographic and clinical characteristics of the groups.

	TKA (*n* = 71)	UKA (*n* = 11)	*p*-Value
**Age (years)**	67.7 ± 7.2	59.8 ± 7.0	0.004 ^a^
**Gender**			0.520 ^b^
Female	46 (64.8%)	6 (54.5%)	
Male	25 (35.2%)	5 (45.5%)	
**Operation side**			0.336 ^b^
Left	39 (54.9%)	3 (27.3%)	
Right	32 (45.1%)	8 (72.7%)	
**Surgical Procedure**			0.002 ^b^
DAIR	20 (28.2%)	8 (72.7%)	
Two-stage revision	51 (71.8%)	3 (27.3%)	
**Median Follow-up Time**	45 months	49 months	0.976 ^c^
(IQR)	(30–63)	(27–66)	

DAIR: debridement, antibiotics, and implant retention; IQR: interquartile range (25th–75th percentile); ^a^ Student’s *t*-test; ^b^ Fisher’s exact test; ^c^ Mann–Whitney U test.

**Table 2:** Infection recurrence according to surgical strategy in TKA and UKA patients.

	TKA (*n* = 71)	UKA (*n* = 11)	*p*-Value
**DAIR**	20 (28.2%)	8 (72.7%)	0.002 ^a^
No recurrence	16 (80.0%)	7 (87.5%)	
Infection recurrence (required 2-stage revision)	4 (20.0%)	1 (12.5%)	
**Two-stage revision**	51 (71.8%)	3 (27.3%)	0.002 ^a^
No recurrence	46 (90.2%)	3 (100%)	
Infection recurrence	5 (9.8%)	0 (0%)	

DAIR: debridement, antibiotics, and implant retention; ^a^ Fisher’s exact test.

**Table 3:** Distribution of isolated microorganisms and culture-negative cases in TKA and UKA.

Microorganisms	TKA (*n* = 71) *n* (%)	UKA (*n* = 11) *n* (%)
**Gram-positive microorganisms**		
*Staphylococcus aureus*	17 (25.3)	6 (54.5)
-MRSA	9 (14.1)	1 (9.1)
Coagulase-negative staphylococci	10 (14.1)	3 (27.3)
-MRCoNS	9 (12.6)	0 (0)
*Streptococcus* spp.	5 (7)	0 (0)
*Enterococcus* spp.	3 (4.2)	0 (0)
*Bacillus* spp.	1 (1.4)	0 (0)
**Gram-negative microorganisms**		
**Enterobacteriaceae**		
*E. coli*	3 (4.2)	0 (0)
-ESBL producing	2 (2.8)	0 (0)
*Klebsiella* spp.	2 (2.8)	0 (0)
-ESBL producing	1(1.4)	0 (0)
*Serratia marcescens*	1 (1.4)	0 (0)
-ESBL producing	1 (1.4)	0 (0)
**Non-fermentative Gram-negative bacilli**		
*Pseudomonas aeruginosa*	2 (2.8)	0 (0)
**Other**		
Polymicrobial	2 (2.8)	0 (0)
**Culture Negative**	25 (33.8)	2 (18.2)

MRSA: methicillin-resistant *Staphylococcus aureus*; MRCoNS: methicillin-resistant coagulase-negative staphylococci; ESBL: extended spectrum beta-lactamase.

## Data Availability

The data presented in the study are available upon request from the corresponding author.
